# Homology and linkage in crossover for linear genomes of variable length

**DOI:** 10.1371/journal.pone.0209712

**Published:** 2019-01-03

**Authors:** Adriaan Merlevede, Henrik Åhl, Carl Troein

**Affiliations:** Computational Biology and Biological Physics, Department of Astronomy and Theoretical Physics, Lund University, Lund, Sweden; Pavol Jozef Safarik University in Kosice, SLOVAKIA

## Abstract

The use of variable-length genomes in evolutionary computation has applications in optimisation when the size of the search space is unknown, and provides a unique environment to study the evolutionary dynamics of genome structure. Here, we revisit crossover for linear genomes of variable length, identifying two crucial attributes of successful recombination algorithms: the ability to retain homologous structure, and to reshuffle variant information. We introduce direct measures of these properties—homology score and linkage score—and use them to review existing crossover algorithms, as well as two novel ones. In addition, we measure the performance of these crossover methods on three different benchmark problems, and find that variable-length genomes out-perform fixed-length variants in all three cases. Our homology and linkage scores successfully explain the difference in performance between different crossover methods, providing a simple and insightful framework for crossover in a variable-length setting.

## Introduction

Evolutionary algorithms are a family of computational methods that utilise natural selection for global optimisation on a wide range of problem types. The connection between evolution in computation and nature has been a positive influence on both fields. Evolutionary computation has benefited from innovation inspired by insights in natural evolution since its inception. [1, 2] For evolutionary scientists, algorithms can function as *in silico* models, allowing specific aspects of evolution to be isolated and studied from a different perspective and with a level of control that is not possible in nature [3, 4].

Genetic information in computational evolution is traditionally represented as an array of genes, in the form of bits or real numbers. [5] In a typical genetic algorithm, genes are read in order from left to right and used as a list of arguments to evaluate the fitness function. [2] Despite being inspired by nature, this is in stark contrast with natural genomes. The genetic material of natural organisms is stored in long polymeric biomolecules (DNA), which may be reorganised or resized due to mutations and imperfect recombination events. In nature, genes are not identified by position, but by context: signalling sequences allow a decentralised system of proteins to recognise and decode genes regardless of where they are located on the DNA double-strand [6].

As a consequence, some types of genotypic variation that are common in natural genomes rarely appear in computational settings, including phenomena such as copy number variations, where genes or longer sequences are duplicated or removed, and structural variations, where sequences move to different locations across the genome. This has potentially far-reaching consequences for the dynamics of the evolutionary process. For example, gene duplications are crucial for the emergence of new genes in natural evolution [7], and this mechanism is intimately linked to genetic properties such as robustness, evolvability, and functional specialisation. [8, 9] For computational modelling of evolution, variable-length genomes therefore not only match the data structure of DNA more closely in a superficial sense, but may also provide a natural way to replicate some interesting and relevant phenomena from evolutionary biology. In a longer perspective, self-organization of genomes is a natural step towards richer and more open-ended models of evolving systems [10].

From the perspective of optimisation, genomes with variable length are a natural choice when the complexity or dimensionality of solutions is itself an unknown to be adapted to the problem. Solutions for encoding a variable dimensional complexity in a fixed-length genome exist [11–13], but allowing the solution size to evolve can also lead to a more natural exploration of the search space; consider a small computer program gradually acquiring more features, as opposed to starting out with a static amount of initial nonsense instructions. Variable-length genomes have been particularly successful in genetic programming [14], grammatical evolution [15] and for evolving neural network topologies [16]: applications where the solution complexity is intended to gradually evolve just like other genetic information. Most genomes of variable length are tree structures, but linear genomes have been used as well [17, 18].

Here, we focus on defining sensible and effective crossover operators for variable-length linear genomes. We conceptually reevaluate recombination in this setting, and define two new numerical scores that measure the ability of a crossover method to reshuffle variant information in the two parents (linkage score) while maintaining structural similarity (homology score). We use these scores to review and compare existing crossover methods, in addition to introducing two novel crossover algorithms. Based on three different benchmark problems, we confirm that the two attributes represented by our scores successfully explain differences in the ability of the crossover methods to accelerate evolution. In all three benchmark problems, optimisation with variable-length genomes out-performs constant-length variants.

## Methods

**Fig 1:**
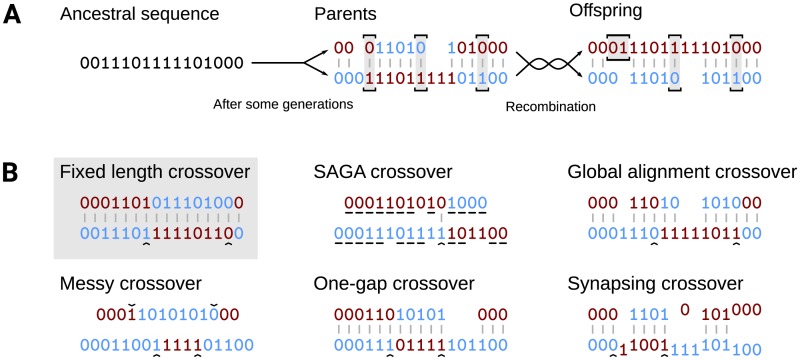
Illustration of crossover and homology and linkage scores with binary genomes. A: Homology and linkage scores. The two sequences are aligned to show homologous pairs, and unequal pairs are highlighted. Bits are coloured by the offspring that inherits them in an example recombination. Homology: The parents share 14 homologous bits, but only 13 pairs remain in the offspring. The homology score is 13/14. Linkage: Out of the homologous pairs that are recombined evenly, 2 consist of unequal elements. The sequence formed by these pairs in the parent genome flips colours 1 time, out of a maximum of 1. The linkage score is 1. B: Schematic representation of different crossover methods. One example two-point crossover is shown for each method, or one-point in the case of SAGA. Randomly chosen points are indicated with a caret (^); alignments are shown where applicable. In the fixed-length example, the sequences have not been affected by insertions and deletions. Underlined bits in SAGA are the longest common subsequence. Global alignment represents both the Hirschberg algorithm and the approximate heuristic alignment.

In linear genomes of constant size, crossover is performed by copying two parental genomes, taking “crossover points” uniformly from across the length of the genome, and exchanging the sequences in between (see Fig 1B). [5] The most popular operators differ only in the number *n* of crossover point pairs they use: *n* = 1 for one-point crossover; *n* = *k* for *k*-point crossover. Uniform crossover, in which each bit is inherited independently from either parent with a probability of 50%, is equivalent to an *n*-point crossover variant, where *n* is drawn randomly from a binomial distribution with a number of trials equal to the length of the genome and a success probability of 50% per trial.

This scheme cannot be applied directly to genomes of different lengths: crossover requires one or more pairs of points, but there is no obvious pairing between sequences of different lengths. In both DNA and fixed-length genomes, the parts that are exchanged during crossover are different variations of the ‘same’ sequence, e.g. different alleles of the same gene. This ‘sameness’—the fact that similar sequences can be found in similar locations in different genomes—is a direct result of the common ancestry of those sequences, which biologists call **sequence homology**. [19, 20] Homologous sequences are typically similar but not identical, having diverged due to mutation after the separation of their ancestral lines. In constant-length genomes, every element is in the same position in every generation, so that homology is unambiguously declared by position within the sequence. In nature, crossover is guided towards homologous sequences by complicated machinery inspecting chromosome organisation and proteins associated with the DNA. [21] Some authors have tagged their variable-length genomes with metadata to guide crossover to sensible locations [22, 23], but generally we have to infer homology from sequence similarity [19, 20].

We define here the **homology score** to capture a crossover’s ability to find and exchange homologous features and thus retain genome structure. Formally, the homology score is the expected fraction of homologous elements in the two parents that are evenly distributed across the two offspring. Crossovers that unevenly distribute homologous parts of the two parent genomes essentially cause insertion and deletion errors in the offspring, and operators that do this often are defined to have a low homology score. The score is dependent on the divergence of the two genomes being crossed, as it is harder to successfully identify homology in genomes that are less similar to each other, and to their common ancestor.

Avoiding uneven recombinations is a goal that would be perfectly accomplished without crossover. The defining feature of recombination is that it redistributes the parents’ genetic information. [2, 5, 6] We introduce the **linkage score** to measure the uniformity of information exchange during recombination. Considering all the homologous pairs that are unequal in the two parents, we define the linkage score as the fraction of consecutive pairs in the parent genomes that are passed on to the offspring in an opposite manner, i.e. that have an odd number of crossover points between them. Only pairs that are distributed to both offspring are taken into account. Both the homology and linkage scores are illustrated in Fig 1A.

The linkage score derives its name from the closely related concept of genetic linkage in biology, which is the correlated inheritance of two genes or sequences due to proximity on the chromosome. Genetic elements that are close together are more likely to be inherited together, because the probability of crossover occurring in the space between them is lower. [6] In a computational setting, then, this correlation of inheritance can be controlled by the number of crossover points, *n*. It is maximal when *n* = 0, i.e. when there is no exchange of genetic information. In the opposite extreme, there is no correlation, such as in the case of uniform crossover. [5] The linkage score as defined here differs from the traditional idea of genetic linkage because it only considers the inheritance correlation of unequal elements. A low linkage or inheritance correlation corresponds to a high linkage score.

The two recombination scores form a dual pair: the homology score measures conservation of identical information, while the linkage score measures redistribution of variant information. There is a natural and expected trade-off between these two goals, as more intense reshuffling of genetic material also provides more opportunity for errors in exchanging homologous parts.

### Alignment

In practice, detailed information on the history of each genetic element is not available during an evolutionary process. In a fixed-length setting, homologous pairs are those which share the same location in the genome, but variable-length crossovers must use heuristics to identify homology. They have this in common with computational biologists: when dealing with data from biological organisms, in the form of DNA, RNA or protein sequences, the identification of homologous substrings is a very common problem [19, 20].

In bioinformatics, alignment is a ubiquitous solution to the problem of inferring homology from sequence information. [19, 20] Alignment algorithms line up two sequences by inserting ‘gaps’, so that each character is partnered with either a gap or a character in the other sequence. This is done in such a way that the similarity between the partnered sequences is maximised, while avoiding excessive gaps. Regions which align well and show a statistically unlikely level of similarity and likely evolved from the same ancestral sequence [19, 20].

Two types of alignment are in widespread use in bioinformatics. Local alignment identifies and aligns only the parts of the given sequences that have the highest similarity (lowest penalty score), ignoring the rest. This is useful when only a part of the sequences is expected to be homologous, or when homologous parts are not necessarily in the same order on both sequences. Alternatively, global alignment algorithms attempt to align two (or more) sequences from start to finish, pairing up as many characters as possible and introducing gaps only when it decreases the overall alignment penalty score [19, 20].

Global alignment is fundamentally related to variable-length crossover, since both attempt to determine which parts of two sequences are most similar, and thus most likely homologous. Different alignment algorithms have been used for crossover. We define an alignment-based crossover as any operator that draws its crossover locations randomly from the paired points generated by an alignment algorithm. The traditional 1-point, *n*-point and uniform crossovers for fixed-length genomes are all alignment-based crossovers, based on a trivial alignment where bits are paired based on shared position index.

### Crossovers

Below we review existing crossover methods for linear variable-length genomes. We avoid distinguishing crossovers that are deemed ‘homologous’, a common theme in literature. [24–26] There is no guarantee that so-called homologous methods exchange homologous sequences, and indeed we show below that in some situations they are less likely to correctly identify homology than simpler methods (see Results). In literature on genetic algorithms as well as other fields, the concept of homology has a long history of erroneous usage [27], often referring to similarity of sequence, function, or location, rather than shared ancestry. [24, 26] Here, we divide the operators into alignment- and non-alignment based groups.

#### Global alignment crossover (Hirschberg or Needleman-Wunsch)

These two dynamic programming algorithms both identify the optimal global alignment of two sequences, by minimising a penalty score. [28, 29] The penalty score is computed as the sum of scores of each aligned pair, plus a penalty for each gap based on its length. Both algorithms are exact and thus give equivalent results, but differ in memory complexity. The global alignment crossover exchanges genetic information between crossover pairs that are randomly chosen among the aligned pairs of the two parental genomes. In genetic programming literature, this crossover is known as maximum homologous crossover [26].

Due to its use in bioinformatics, global alignment is implemented in libraries for many languages. The alignment also results in a penalty score, which can be used as a measure of similarity between organisms. This is useful in some applications of evolutionary computation, for example when applying niching, a technique that increases genetic diversity in the population by restricting sexual crossover to organisms that are sufficiently similar [30].

We use here a Hirschberg implementation with a penalty of −1 for each matching pair, +5 for each mismatch, and an affine gap penalty of 20 to open and 3 to extend. These parameters were selected as suitable for binary strings.

#### Global alignment crossover (Heuristic)

Unfortunately, the computational difficulty of the Needleman-Wunsch and Hirschberg algorithms is proportional to the product of the lengths of the parent sequences. This may make exact global alignment impractical, depending on the typical length of the genomes and the computation time of the fitness function. While many heuristic solutions can be found in literature that are optimized for different scenarios in bioinformatics [31, 32], we propose here a new method which is optimized for sequences with very high sequence similarity, as they are likely to be encountered in the context of crossover for evolutionary computation.

Our proposed heuristic is a divide-and-conquer approach, fixing a small portion of the alignment and then recursively continuing to the two smaller alignment problems on the left- and right-hand sides of it. Given two sequences of at least 128 bits, the algorithm aligns a random substring of 64 bits from one genome with the most similar candidate on the other genome, on the condition that there are at most 20 mismatched bits, and at least 4 mismatches fewer than the next best match. These stringent requirements ensure that the heuristic only aligns parts that likely belong together in the correct global alignment, despite not considering the context of the whole genomes. If the conditions are not met, the algorithm retries with another random substring up to four times. The remaining sequence pairs are aligned with the Hirschberg algorithm.

#### One-gap alignment crossover

We propose here another novel approach to variable-length crossover, combining the conceptual clarity of alignment-based methods without the computational complexity of examining sequence similarity. The one-gap approach aligns two parental genomes simply by inserting a single gap at a random location on the shorter sequence. The gap is the same size as the difference in lengths between the two parental genomes.

The one-gap crossover has several desirable properties, mainly that it is easy to implement and fast to run. It is also well-behaved with respect to sequence length, as offspring generated with the one-gap alignment crossover always have the same length as one of the parent sequences. Among the methods presented here, it is unique in that it reduces to the traditional fixed-length crossover when the two parental genomes have the same length. This method is similar to the ‘homologous crossover’, or sticky crossover, from genetic programming [24].

Several approaches have previously been proposed for recombining genomes of different lengths, that are not explicitly based on alignment. These methods are summarised below. If possible, we rephrase or generalise them to use any number of crossover points, *n*. Each of these methods is schematically represented in Fig 1B.

#### Messy crossover

The simplest solution to the problem of pairing up crossover points is to take random points on both genomes. We call this method messy crossover because it is based on the approach originally implemented in the messy genetic algorithm [33], but it has also been used in fields such as genetic programming. [18, 22] It results in highly asymmetric recombinations. In genetic programming, this is typically mitigated by controlling the distance between consecutive crossover points [18].

We generalise the messy crossover to an *n*-point method by drawing a sorted list of *n* points from a uniform distribution (without replacement) on each parent genome to use as crossover points. Since the messy crossover will recombine any two points, it can be thought of as maximising the linkage score. Conversely, it pays no regard to homology.

#### SAGA and VIV crossovers

At least two published methods choose a random point on one parent genome, and attempt to identify the most sensible match on the other genome, based on sequence similarity of the surrounding region. In the case of the SAGA cross, the algorithm maximises the similarity between the parts of the parental genomes to the left of the crossover points, plus that to the right. The similarity measure used for the two parts is the longest common subsequence; [34] note that this is distinct from the longest common substring, as a subsequence is not necessarily contiguous. The VIV crossover compares a fixed window around the chosen point with all similarly sized windows on the partner genome, and chooses a partner point randomly in the window that is most similar [35].

Because of the similarity between these methods, we investigate only the more sophisticated SAGA cross. The approach is costly to run [34], and we do not generalise it to use arbitrary *n* crossover points.

#### Synapsing crossover

The Synapsing Variable-Length Crossover is a crossover method inspired by the chiasmata that form during meiosis in biology. [36] It attempts to identify the longest common substring of the parent genomes, which is tagged as a ‘synapse’. After finding a synapse, the procedure is repeated on the left-hand side as well as the right-hand side of the synapse, until the longest common substring is smaller than a threshold value. A random selection of the synapses is then used as crossover points.

This method is capable of *n*-point crossover, without modification. The implementation used in this work uses a minimum synapse length of 10 bits.

We note that the synapsing crossover can also be seen as an alignment-based crossover, since it generates a list of paired points from which to pick a crossover pair. In addition, both the synapsing and SAGA crossovers implicitly use alignment as part of their logic. Synapsing crossover identifies synapses by repeatedly finding the longest common substring, which is a special case of local alignment with an infinite gap penalty. The SAGA cross uses the length of the longest common subsequence to measure the similarity of two substrings, which is equivalent to a global alignment score with no gap penalty. Both these algorithms can be elegantly generalised to use other alignment schemes. We do not implement this here. To our knowledge, it has not been proposed elsewhere.

Below, we also performed each experiment with a cloning operator, which is a trivial crossover that copies one of the parents with no recombination. This is a control method, exchanging no information and making no recombination errors.

### Near-uniform crossover

The linkage score of a crossover methods increases naturally with the number *n* of crossover sites, as higher *n* makes it increasingly likely that any two bits are separated by an odd number of crossover points and thus end up on different genomes. In the extreme case, there is no correlation of inheritance between any two bits. This corresponds to a linkage score of 0.5.

Consider that, to have no inheritance correlation, the probability of having an odd number of crossover points between any two bits on the same genome must be exactly 50%. This must also be true for any two subsequent bits, which means that any bit must individually be selected as a crossover point with a probability of 50%, and so the expected value of the total number of crossover points *n* must be half the length of the genome. For fixed-length genomes, this is popularly implemented by uniform crossover. However, when the two parent genomes are different in length, it is impossible for the required conditions to be true for both parents at the same time.

Instead, we define here a **near-uniform crossover**, where the expected number of crossover sites *n* is half of the maximum number of sites. For messy crossover, the maximum number of sites is the length of the shorter parent genome; for alignment-based methods (including synapsing), it is the number of aligned pairs. We draw *n* from a binomial distribution, so that near-uniform crossover applied to constant-length genomes is exactly equivalent to uniform crossover.

## Results and discussion

We first analysed the recombination scores, followed by an analysis of performance in three different benchmark problems. Each method occurs in three variants with different numbers of crossover points: 1-point, 3-point and near-uniform. SAGA was only evaluated as 1-point. All experiments use binary genomes, but the methods generalise naturally to other alphabets.

### Recombination scores

In order to track the evolutionary history of each sequence element, we generate random genomes of length 1000, and tag each bit with a unique identifier. The tags are retained through mutations, so that the complete list of homologous bit pairs is revealed when comparing the tags of a mutated sequence to its ancestor.

The mutation operator is composed of three different mutation types: substitutions, which flip bits in the sequence; deletions, which remove parts of the sequence; and insertions, which make the sequence longer by copying a random section from the genome. Deletions and insertions (indels) affect segments of length *l*, drawn from a power-law distribution proportional to *l*^−2^ (truncated at the end of the genome). A similar power-law distribution for the size of indels has been observed in nature. [37] Each of the three mutation types affected any particular bit with a probability of approximately *p*_*m*_, the mutation strength, which was varied from 0% to 20%. At the highest mutation strength, approximately half of the genome was affected by at least one of the three operators; this is an extremely high sequence divergence.

**Fig 2:**
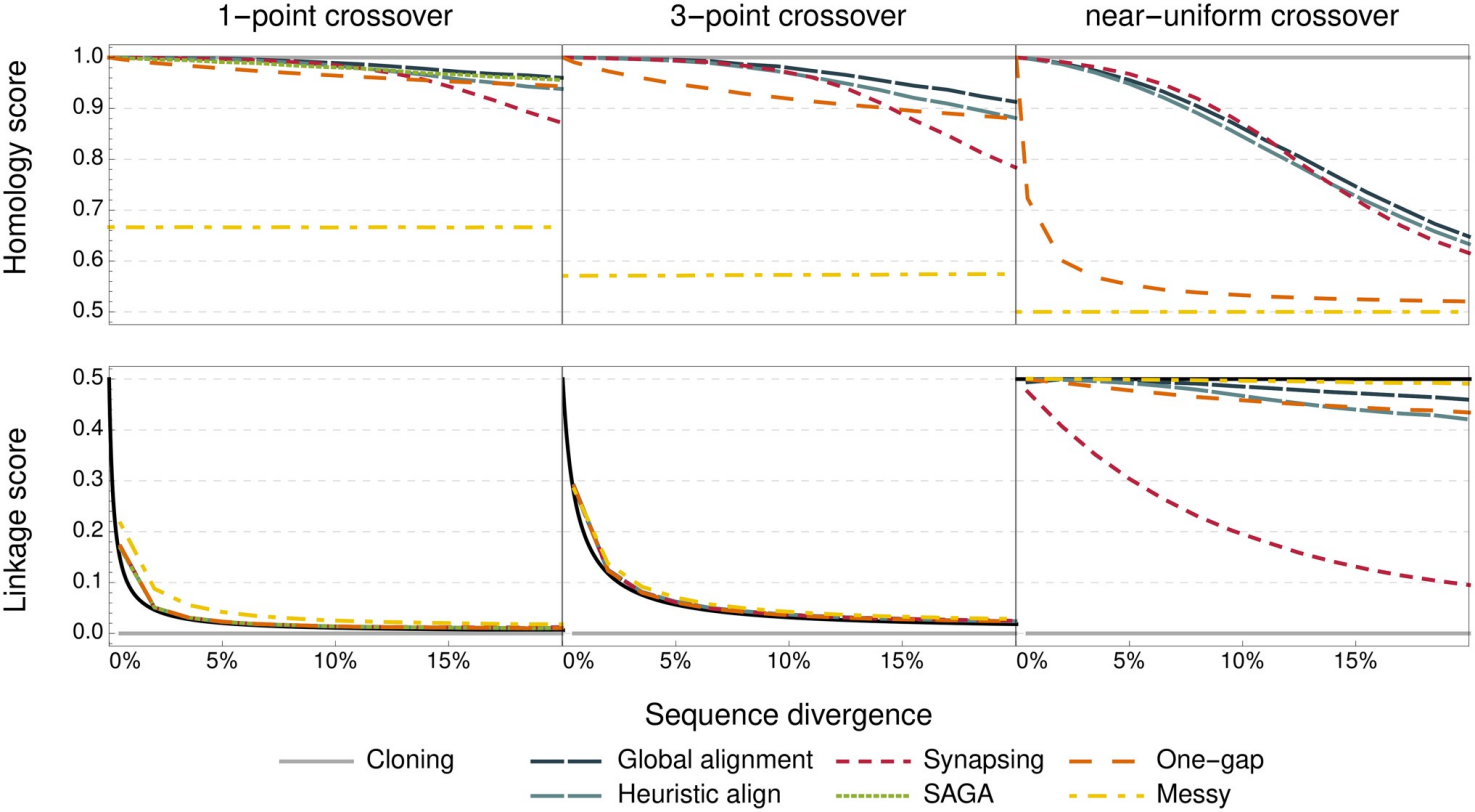
Homology and linkage scores for different crossover operators. The homology score (top) and linkage score (bottom) are shown as functions of the mutational distance between parent sequences for different crossover methods and the cloning control, for different numbers of crossover points (left to right). The black curve shows the linkage score for a fixed-length crossover with an approximately comparable length and divergence. Scores for each operator are means over 10^4^ parent sequences.

The resulting scores, computed as an average of 10000 recombinations of different genome pairs, are shown in Fig 2.

As expected, operators with more crossover points attain a higher linkage score, at the cost of fidelity in preserving homologous information. This trade is much more favourable when the parent sequences are less diverged. Unless the sequences are highly dissimilar, most crossover methods have similar scores. Messy crossover and cloning stand out because they occupy the two extremes in the trade-off between reshuffling variation and retaining homology, maximising one while sacrificing any attempt to achieve the other, regardless of sequence divergence.

The homology score of messy crossover is independent of the sequence divergence, as the choice of crossover points is not dependent on either sequence context or position. At *n* = 1, the non-homologously recombined pairs are those that are to the right of the crossover point on one parent, and to the left on the other parent. These are, on average, $\frac{1}{3}$ of the points, which is the expected size of the interval between two points chosen from a uniform distribution. The near-uniform messy crossover has a homology score of $\frac{1}{2}$, indicating that there is no correlation between the inheritance of homologous bits.

Overall, the exact and heuristic global alignment methods have the highest homology scores among methods with a similar linkage score, and vice versa. Both SAGA and synapsing crossovers perform nearly on par with global alignment. However, these methods cannot navigate the linkage-homology trade-off with the same reach, because they are both limited in the number of crossover points. In the case of synapsing, this is because the alignment it produces does not attempt to cover as much of the genome as possible, allowing crossover at relatively few points even in the near-uniform variant. Synapsing is also consistently unable to separate and recombine certain variations, in particular when the sequence separating them is shorter than the minimum synapse length.

The one-gap crossover is not drastically different from the other methods when using only one crossover point. This is remarkable because the one-gap method does not consider any measure of sequence similarity, and therefore does not qualify as a homologous crossover according to the term’s traditional use. Instead of sequence similarity, it uses only the location of features in the genome to infer homology. Remarkably, the homology score for one-gap even exceeds that of the other methods when divergence is very high.

### Benchmarks

To test the performance of each crossover operator, we performed three benchmark experiments with an evolutionary algorithm. Each benchmark is identical except for the fitness function. The experiments used a population size of 100, an initial genome length of 1000, and mutation strength of *p*_*m*_ = 0.002.

In each iteration of the algorithm, one individual is replaced in the population. The individual to be removed is selected by a small tournament, where two random individuals are chosen and the one with lower fitness is removed. It is then replaced by a new individual, generated either through crossover without mutation (with probability *p*_*x*_ = 0.15) or by copying an existing individual with mutation (with probability 1 − *p*_*x*_). In both cases, each parent is picked as the winner of another tournament. The tournaments are repeated as necessary so that two parents for crossover are always distinct.

The three benchmarks are designed to represent different alleged benefits of crossover.

#### Benchmark 1: string match

In the string match problem, fitness is defined as the similarity between an individual’s genome and a given target string. The similarity itself is measured by the penalty score of a global alignment, using the same parameters as the crossover described above. The target string is a fixed string of 749 bits: the 7-bit ASCII encoding for the sentence ‘*“offensive” is frequently but a synonym for “unusual”; and a great work of art is of course always original*’.

The string match problem is designed to exemplify the ability of recombination to ‘heal’ the effects of minor negative mutations. Mutations with a negative effect on fitness are known to accumulate in populations that reproduce asexually, because their inheritance can be arbitrarily coupled with positive mutations that occur in the same genome. The only way to remove negative mutations is to have a rare back-mutation in the same position. In sexual populations, recombination can separate the different variations so that they can fixate or die out independently [38].

#### Benchmark 2: substrings

For this benchmark problem, the goal is to produce individuals that contain a certain set of target bit sequences as substrings in their genome. There are 1024 randomly generated targets, each with a length of 16 bits. The fitness of a genome is proportional to the number of target sequences that the genome contains as substrings.

The intention of the substrings benchmark is to provide an environment where the main use of crossover is to recombine ‘building blocks’ that evolved in the different lineages of the two parents. The target substrings here can constitute building blocks, but may also form meaningful regions much larger than 16 bits by overlapping. Genomes in this benchmark have no gradual changes in fitness, only the discrete steps of accumulating or losing targets, and there is no direct incentive to form any genome-wide structure.

#### Benchmark 3: RBF

In this benchmark, individuals are trained to approximate a fixed target function as a sum of radial basis functions (RBF). Each gene represents a triangle-shaped RBF of the form $x\mapsto \text{max}(0,(h-\frac{h}{w}|x-x_{0}\left|\right)$, and is recognised in the genome by a tag sequence (110011), similar to the recognition of genes by the presence of short promoter sequences in living organisms. The 30 bits after the tag encode the centre (*x*_0_), width (*w*) and height (*h*) of the RBF. The numbers are read consecutively, each decoded from 10 bits to a number using the binary base number system, and rescaled linearly from [0, 1023] to [0, 1), (0, 0.5] and [−1, 1], respectively. Finally, the sum *f* of all the RBFs in the individual’s genome is compared to the target function *f*_0_ = sin(12*πx*) by computing the error integral $fitness=-\int_{0}^{1}{\left(f\left(x\right)-f_{0}\left(x\right)\right)}^{2}dx$.

RBF is a problem where variable-length genomes are a natural representation, and where evolution should benefit from crossover in several ways.

A well-known issue with genomes of variable length is the uncontrolled growth of genome size, called “bloat” or “fluff”. [18, 39] To manage genome size, we multiply the fitness function with an additional length-dependent penalty factor for genomes larger than 1000 bits. In the case of the substrings problem, which has positive fitness values, we use a factor equal to 2 − *l*/1000, where *l* is the length of the genome. For the RBF benchmark, which has negative fitness values, we multiply the fitness by *l*/1000. The string match benchmark is self-regulating with respect to genome size, since the fitness function favours sequences with a similar length to the target. Many other solutions exist for limiting bloat. [18, 39] Our method was chosen for simplicity. It introduces a steep fitness penalty, effectively culling genome sizes exceeding our threshold 1000 by more than a few bits.

**Fig 3:**
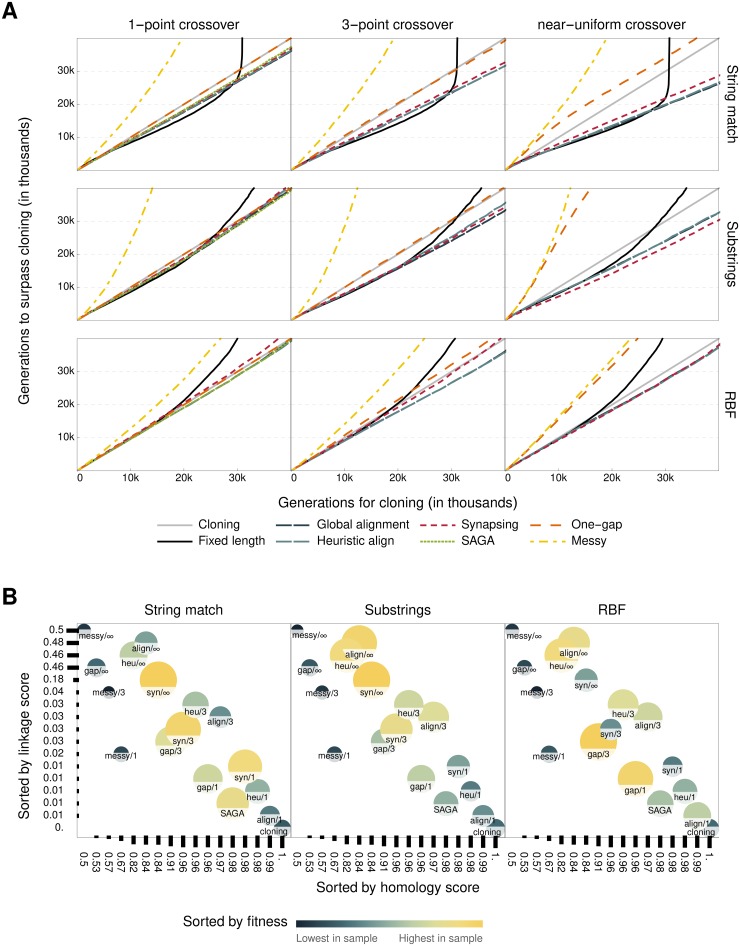
Benchmark performance and connection to recombination scores. A: Performance of each crossover method (line colour) for each problem (row) and number of crossover points (column). The curves show the number of generations needed to surpass the final fitness in a control experiment (with Cloning) of a given duration. B: The fitness at the end of each experiment (colour and size) in relation to the recombination scores (axes). Each variable is shown by rank instead of absolute value, in order to maximise visibility of robust patterns. Recombination scores were measured at *p*_*m*_ = 0.11, but the same patterns are visible at other mutation strengths.

We ran each experiment with each of the different crossover methods, in order to compare their influence on the rate of evolution. The fitness at each generation is that of the best individual, averaged over many runs (*n* = 400, 100, 1000 respectively for the three benchmarks) after discarding the 50% runs with the lowest final fitness. The results are shown in Fig 3A, and are related to the recombination scores in Fig 3B. S1 Code contains C++ source code for the crossover algorithms, recombination scores and evolution benchmarks.

#### Crossover accelerates evolution based on its recombination scores

Crossover helped populations evolve faster towards the target in all three benchmarks (Fig 3A). However, not all problems are equally dependent on recombination for efficient evolution, with crossover having the least crucial role in the RBF benchmark. Correspondingly, the choice of crossover does not have the same influence on the rate of evolution. Crossovers with very low scores slow down the evolutionary process, compared to cloning.

There is a strong relation between the recombination scores and performance (Fig 3B). In order to maximise the efficiency of an evolutionary optimisation, high fidelity is necessary with respect to preserving homologous information. However, this feature of crossover is common, and can be achieved even by simple algorithms such as one-gap recombination. Provided the homology score is high, performance of the different crossovers is best explained by the linkage score. In both the string match and RBF benchmarks, operators with a higher linkage score perform better, even though it comes at a small cost to fidelity. Notably, the global alignment crossover is the most effective in these cases, not because it maximises the correct inference of homology, but because its near-uniform variant has the highest linkage score. This result is unexpected.

In the substrings problem, the best performing operator is near-uniform synapsing, with an intermediate linkage score (Fig 3A and 3B). We hypothesised that this problem may evolve faster when segments of a certain length are more likely to be inherited together. Indeed, the substrings benchmark is intended to exemplify the mechanism of increasing fitness by exchanging individually useful building blocks, which is facilitated by a crossover with a low number of crossover points in genomes with fixed length. Alternatively, it is possible that the effectiveness of near-uniform synapsing is caused by an evolutionary mechanism that is not measured in our artificial scoring environment. For example, it is conceivable that the population adapts certain genomic features that guide the synapsing algorithm to cross over in places that are more likely to result in fit offspring. In order to distinguish between these two hypotheses, we repeated the experiment with global alignment using between 5 and 50 crossover points. This confirmed that variants of the global alignment crossover with an intermediate number of crossover points could perform on par with near-uniform synapsing.

#### Variable-length genomes evolve faster than fixed-length genomes

To compare the performance of variable-length genomes to fixed-length ones, we performed the same experiments without insertion and deletion mutations, and using traditional *n*-point crossover. The crossover used was a traditional *k*-point and uniform crossover. The genomes were fixed to 1000 genes in length, or 749 bits in the case of the string match benchmark.

In all three benchmarks, variable length representation and indel mutations increase the optimisation performance of the evolutionary algorithm (Fig 3A). This is remarkable, especially because there is little change in genome length during the experiment due to the stringent length-stabilising selection. We attribute this observation to the fact that our fitness functions do not interpret bits directly by their position within the genome, so that successful substrings do not have to evolve in any particular location. Indel mutations allow substrings to move around by deleting gaps in between them, effectively defragmenting the genome. Without indels, populations evolve faster initially because there is less disruption from mutations, but get stuck in local optima when they cannot optimise the relative locations of successful substrings.

#### Effect of crossover on optimal mutation rate

It has previously been reported that using crossover increases the optimal mutation rate in fixed-length genomes. [40] Crossover diminishes the disruptive effect of mutations by decreasing the accumulation of deleterious mutations [38], and neutral mutations can become beneficial when recombined into a different background [40].

**Fig 4:**
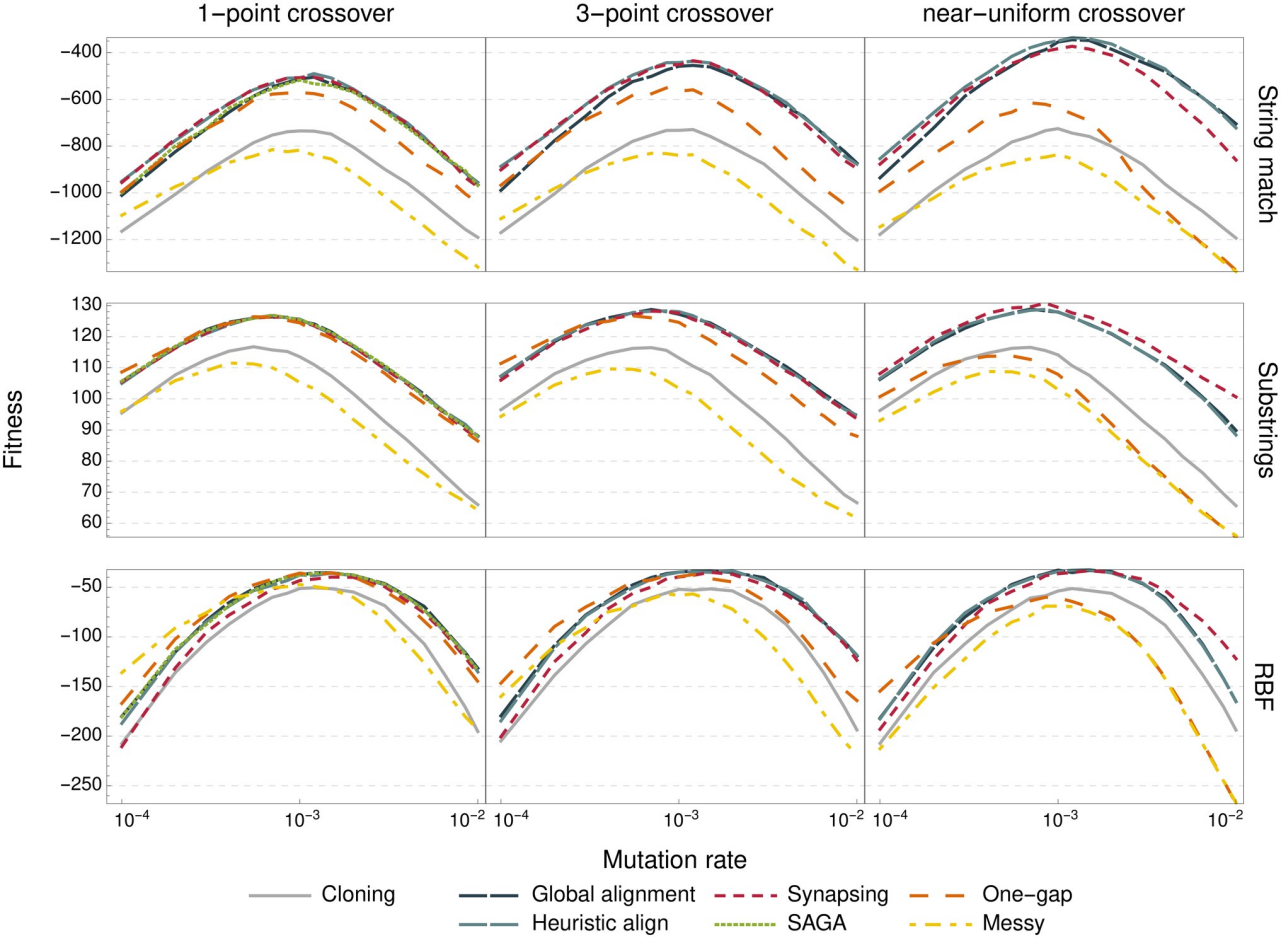
Mutation rate sensitivity. Final fitness value after 20000 generations for each operator (line colour), as a function of the mutation rate, *p*_*m*_ (logarithmic scale), for each problem (row) and number of crossover points (column). All other parameters were unchanged.

To expand upon this, we repeated our performance experiments for different mutation strengths, as shown in Fig 4. We found that using crossover indeed increases the optimal mutation rate. Moreover, crossovers with a better performance also tend to have a higher optimal mutation rate, although the relationship is not absolute (Spearman correlation: *ρ* = 0.88; *p* < 10^−5^ (string match); *ρ* = 0.78; *p* < 10^−3^ (substrings); *ρ* = 0.47; *p* = 0.054 (RBF)). We hypothesised that crossovers which are better capable of separating mutations (linkage score) may mitigate the damages of high mutation rate by filtering out deleterious mutations quicker. Alternatively, it is possible that crossovers which are better at meaningfully recombining more different parents (homology score) are less disrupted by the diversity that comes with higher mutation. However, neither of the two recombination scores were significantly correlated with the optimal mutation rate.

We also observe that crossovers with low scores, which had very low performance at nominal mutation levels, can outperform the negative control and even other crossover methods when mutation rate is very low. This may indicate that lowering the mutation rate leads to a regime where crossover is primarily responsible for creating new variation, rather than recombining existing variation. In other words, it suggests that non-homologous crossovers can partially replace mutation as a source of variation in an evolutionary process.

## Conclusions

Our work presents a new look at crossover in variable-length linear genomes, highlighting the dual importance of correctly exchanging homologous information, and recombining unique variations in the two parents. We quantify these two goals by defining the homology and linkage score, which can be measured for any crossover operator and sequence divergence, and show that these two factors indeed explain the difference between crossover methods. The trade-off between linkage and homology is controlled in large part by the number of crossover points. In the extreme case, we provide an approximation of uniform crossover for variable-length genomes by defining the near-uniform crossover.

While both homology and linkage are important for successful recombination, our results show that, given a method that is reasonably effective at recognising homology, the most crucial factor for efficient evolution is choosing an appropriate correlation between the inheritance of genomic variations. The global alignment crossover allows the most flexible tuning of the linkage score by working with a wider range of crossover points. In particular, it has the highest number of possible crossover points among the reliable methods, and its near-uniform variant approaches the uniform linkage score.

In many cases, desirable crossovers do not only accelerate evolution, but are also easy to implement and fast to compute. The one-gap crossover we presented here is very simple and easy to run, making it the most efficient algorithm in terms of programmer time, while requiring between 10% and 50% more fitness evaluations to evolve the same solution, compared to the best method for each problem. We also propose a heuristic alternative to the Hirschberg algorithm for global alignment, which runs 3–4 times faster than the exact method in our experiments, with virtually no difference in performance for our scores and benchmarks. There is likely more room for improvement for heuristic alignment methods that are optimised for use in evolutionary computation.

Our work looks at crossover in evolutionary algorithms with linear genomes of variable length. In the future, it may be interesting to investigate the theoretical goals and practical implementation of crossover for genomes which may not only change in length, but also in structure, by allowing parts of the genome to be moved or copied.

## Supporting information

S1 Code Crossover source code. Source code of the C++ program used to generate all data.
(ZIP)
